# Macrogeographic genetic structure of *Lutzomyia longipalpis* complex populations using Next Generation Sequencing

**DOI:** 10.1371/journal.pone.0223277

**Published:** 2019-10-03

**Authors:** Aline Etelvina Casaril, Diego Peres Alonso, Karina Garcia Franco, Marcus Vinicius Niz Alvarez, Suellem Petilim Gomes Barrios, Wagner de Souza Fernandes, Jucelei de Oliveira Moura Infran, Ana Caroline Moura Rodrigues, Paulo Eduardo Martins Ribolla, Alessandra Gutierrez de Oliveira

**Affiliations:** 1 Programa de Pós-Graduação em Doenças Infecciosas e Parasitárias, Universidade Federal de Mato Grosso do Sul, Campo Grande, Mato Grosso do Sul, Brasil; 2 Laboratório de Parasitologia Humana, Universidade Federal de Mato Grosso do Sul, Campo Grande, Mato Grosso do Sul, Brasil; 3 Laboratório de Pesquisas e Análises Genéticas, Departamento de Parasitologia, Universidade Estadual Paulista, Botucatu, São Paulo, Brasil; 4 Laboratório de Doenças Parasitárias, Universidade Estadual do Ceará, Fortaleza, Ceará; Instituto Oswaldo Cruz, BRAZIL

## Abstract

*Lutzomyia longipalpis* is the main vector of *Leishmania infantum*, the causative agent of visceral leishmaniasis in the Neotropical realm. Its taxonomic status has been widely discussed once it encompasses a complex of species. The knowledge about the genetic structure of insect vector populations helps the elucidation of components and interactions of the disease ecoepidemiology. Thus, the objective of this study was to genotypically analyze populations of the *Lu*. *longipalpis* complex from a macrogeographic perspective using Next Generation Sequencing. Polymorphism analysis of three molecular markers was used to access the levels of population genetic structure among nine different populations of sand flies. *Illumina Amplicon Sequencing Protocol*^®^ was used to identify possible polymorphic sites. The library was sequenced on paired-end Illumina MiSeq platform. Significant macrogeographical population differentiation was observed among *Lu*. *longipalpis* populations *via* PCA and DAPC analyses. Our results revealed that populations of *Lu*. *longipalpis* from the nine municipalities were grouped into three clusters. In addition, it was observed that the levels of *Lu*. *longipalpis* population structure could be associated with distance isolation. This new sequencing method allowed us to study different molecular markers after a single sequencing run, and to evaluate population and inter-species differences on a macrogeographic scale.

## Introduction

The history of visceral leishmaniasis (VL) in the Americas is closely related to the *Lutzomyia longipalpis* species, which was described by Lutz and Neiva [1] and identified as a *Leishmania infantum* vector based on consistent evidence from various studies on sand fly vector competence [2, 3, 4, 5, 6].

Brazil has a high prevalence of VL among the Neotropical countries [7] probably due to the adaptability of the vector, which presents a wide geographic distribution and occurs in all regions of the country [8, 9, 10].

The taxonomic status of *Lu*. *longipalpis* has been discussed since the late 1960s, when Mangabeira [11] observed that males collected in the States of Ceará and Pará differed in the number of spots present in the abdominal tergites. Subsequent studies showed that different Brazilian populations of *Lu*. *longipalpis* produced different pheromones among themselves and presented reproductive isolation [12, 13, 14, 15, 16]. These findings support the hypothesis that *Lu*. *longipalpis* represents a complex of species, which could reflect into the different epidemiological profiles of the disease, once sand flies present specific habits, behaviors and capacity of infection.

The advent of high-throughput genotyping, referred to as Next Generation Sequencing, made it possible to analyze molecular markers on a large scale and on a huge number of individuals, revealing this technique as an excellent tool for population genetics studies. Among the numerous molecular markers used for entomological studies, the simultaneous sequencing of the nuclear and mitochondrial genomes is shown to be more reliable because it reveals different evolutionary events [17, 18, 19, 20, 21, 22].

In this study, we used Next Generation Sequencing (Illumina MiSeq platform) to analyze populations of the *Lu*. *longipalpis* complex from different Brazilian locations, based on the polymorphisms detected in two nuclear regions (period gene, IVS6 of cacophony gene) and in the mitochondrial 12SrDNA ribosomal region.

## Materials and methods

### Ethics statement

A permanent license for collecting and transporting zoological material (Protocol 25592–1) was obtained on the behalf of PhD. Alessandra Gutierrez de Oliveira, issued by the System of Authorization and Information on Biodiversity of the Brazilian Institute of Environment and Renewable Natural Resources (Sisbio/IBAMA).

### Study area and period

**Fig 1:**
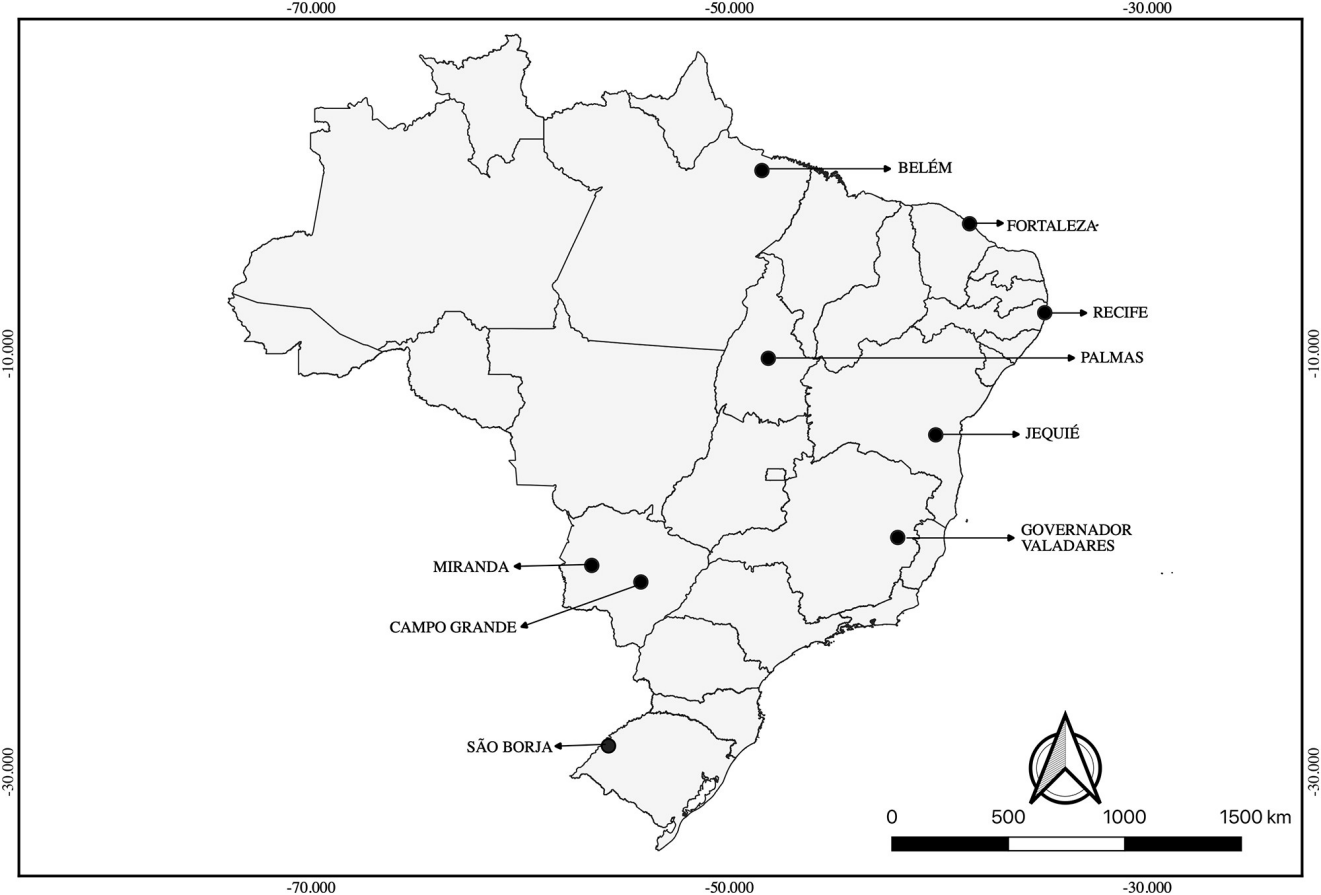
Geographic distribution of *Lutzomyia longipalpis lato sensu* specimens collected, Brazil, 2017.

Sand flies were captured between August 2014 and December 2016 from nine municipalities described in Fig 1. Collections were performed using light traps (Falcão modified). All sandflies were identified based on morphological characteristics of the genitalia, head, and thorax, as described by Galati [23]. We observed the expected pattern of tergite spots in *Lu*. *longipalpis* individuals (Table 1).

**Table 1:** Collection sites and number of spots in the abdominal tergites in *Lutzomyia longipalpis* from different Brazilian regions and biomes, Brazil (n = 45).

Species	Municipality	State	Spot	Brazilian Region	Biome	N
*Lu*. *longipalpis*	Belém	PA	1S	North	Amazonia	5
*Lu*. *longipalpis*	Campo Grande	MS	1S e 2S	Central-West	Savanna-like Cerrado	5
*Lu*. *longipalpis*	Fortaleza	CE	2S	Northeast	Caatinga	5
*Lu*. *longipalpis*	Governador Valadares	MG	1S	Southeast	Atlantic Rainforest	5
*Lu*. *longipalpis*	Jequié	BA	1S	Northeast	Caatinga and Atlantic Rainforest	5
*Lu*. *longipalpis*	Miranda	MS	1S	Central-West	Pantanal wetland	5
*Lu*. *longipalpis*	Palmas	TO	1S	North	Savanna-like cerrado	5
*Lu*. *longipalpis*	Recife	PE	2S	Northeast	Atlantic Rainforest	5
*Lu*. *longipalpis*	São Borja	RS	1S	South	Pampa	5
**TOTAL**						45

N: number of specimen; PA: Pará; MS: Mato Grosso do Sul; CE: Ceará; MG: Minas Gerais; BA: Bahia; TO: Tocantins; PE: Pernambuco; RS: Rio Grande do Sul.

### Extraction of DNA

After morphological identification, five male insects from each study locality were preserved in 70% alcohol and subsequently crushed using a plastic pestle and portable mixer in 1.5 mL tubes containing 300 μL of 5% Chelex^®^ Molecular Biology Grade Resin (Bio-Rad Laboratories, Hercules, USA) according to the manufacturer’s recommendations. The solution was vortex-mixed for 15s and subsequently centrifuged for 20 s at 11,000 g. Next, the solution was placed in a water bath at 80°C for 30 min, and then the procedure was repeated. The supernatant was removed, transferred to another sterile Eppendorf tube and frozen at -20°C.

### *Polymerase Chain Reaction*—PCR

Illumina Amplicon Sequencing Protocol was used to amplify possible polymorphic sites present on the *Lu*. *longipalpis* from each locality, totaling 45 specimens. PCR was performed using three pairs of oligonucleotides to which we added the sequences 5’ TCGTCGGCAGCGTCAGATGTGTATAAGAGACAG before the *forward* oligonucleotide, and 5’ GTCTCGTGGGCTCGGAGATGTGTATAAGAGACAG before *reverse* oligonucleotide (Table 2). These sequences are required for the hybridization of each amplicon in the Illumina flowcell.

**Table 2:** Sequences of oligonucleotides and conditions for sand fly DNA amplification.

Region	Primer sequence (5’-3’)	Amplification conditions	References
**12SrDNA**	T1B/T2A TCGTCGGCAGCGTCAGATGTGTATAAGAGACAGAAACTAGGATTAGATACCCT GTCTCGTGGGCTCGGAGATGTGTATAAGAGACAGAATGAGAGCGACGGGCGATGT	94°C for 5 min 35 cycles of 94°C for 20 sec 54°C for 30 sec 72°C for 25 sec 72°C for 5 min	Adapted from Beati *et al*. [24]
**IVS6 –cacophony**	3Llcac/5LIcac TCGTCGGCAGCGTCAGATGTGTATAAGAGACAGGTG GCCGAACATAATGTTAG GTCTCGTGGGCTCGGAGATGTGTATAAGAGACAGCCACGAACAAGTTCAACATC	94°C for 12 min 35 cycles 94°C for 30 sec 55°C for 30 sec 72 °C for 30 sec 72°C for 10 min	Adapted from Lins *et al*.[25]
***Period***	5L1per1/3L1per1 TCGTCGGCAGCGTCAGATGTGTATAAGAGACAGCAATGGCTTCTACATCACTC GTCTCGTGGGCTCGGAGATGTGTATAAGAGACAGACTTGCTGCTTCACTGTATC	95°C for 3 min 95°C for 30 sec 55°C for 30 sec 72°C for 1 min 72°C for 5 min	Adapted from Bauzer *et al*. [18]

#### Sand fly mitochondrial 12S rDNA amplification and sequencing

PCR amplification of *Lutzomyia* sp. 12S rDNA mitochondrial region was performed with the primers T1B and T2A, according to Beati et al. [24].

#### Sand fly nuclear DNA amplification and sequencing

To analyze the polymorphisms in nuclear DNA, we used: Llcac and 5LIcac for the region IV6S cacophony [25] and 5L1per1 and 3L1per1 for the period region [18].

Twenty-five μL of PCR reactions were prepared as follows: 14.1 μl of ultrapure water, 2.5 μl of Buffer 10x, 0.4 μl of dNTPs (0.1 mM), 0.75 μl of MgCl_2_ (50 mM), 1 μl of each oligonucleotide (10 pm/μL), 0.25 μL of TaqDNA polymerase (Ludwig Biotec^®^ PCR kit) and 5 μl The design of the oligonucleotides and the conditions of each amplification are shown in Table 2.

### DNA sequencing

Five specimens from nine different municipalities were analyzed (5x9 = 45 specimens). Each specimen was amplified to the period gene, IVS6 from cacophony gene and 12SrDNA (45x3 = 135 PCR amplicon). The product of the 3 amplified from each individual was pooled in a single tube, totaling 45 pools. These samples containing the amplicons were purified with AMPure XP beads at 1.80x total volume. An index pair (P5 and P7) Nextera^®^ Index Primers (Illumina, San Diego USA) was added for each sample through a PCR with limited cycles. The conditions for Nextera^®^ PCR indexing were: initial denaturation at 95°C for 3 minutes, followed by 12 cycles of denaturation at 95°C for 30 seconds, annealing at 55°C for 30 seconds and extension at 72°C for 30 seconds, with a final extension at 72°C for 5 minutes. A new purification with AMPure XP beads at 1.80X was performed. Then, the amplicons were quantified using Qubit 2.0 fluorometer following the manufacturer’s recommendations for Qubit dsDNA HS (High Sensitivity, Invitrogen) kit.

Samples with distinct multiplexing indices were combined in equimolar ratios to compose a final library for sequencing. The library quantification was made with KAPA library quantification kit^®^ in a qPCR reaction. The reaction was carried out in a thermal cycler as follows: initial denaturation at 95°C for 5 minutes followed by 35 cycles of denaturation at 95°C for 30 seconds and annealing/extension at 60°C for 45 seconds.

The samples were pooled, normalized and denatured, and finally loaded on the Illumina reagent cartridge. One library was paired-end sequenced in 150-cycles in a *Miseq Illumina*^®^ (Instituto de Biociências de Botucatu, Universidade Estadual Paulista “Júlio de Mesquita Filho”).

### Statistical and structural analyses

Briefly, all sequence reads were quality filtered using the default parameters. Then, each individual’s sequence reads were aligned to the *Lu*. *longipalpis* reference genome using Bowtie with default parameters [26].

Genetic differentiation among subpopulations was estimated by the Wright fixation index (*F*_*ST*_ *pairwise*) [27], using Genepop software version 4.2.

The two matrices (AB) of genetic diversity mean (FST pairwise) and geographic distance (Km) were tested for a linear correlation using the Mantel test [28, 29, 30, 31, 32]. P values were calculated using the correlation coefficient r (AB), estimated for 9,999 permutations. The Mantel test was performed using the Ade4 package of software R v.3.5.1. Geographic distances between locations were determined using the Google Earth Pro program.

In order to infer the number of subpopulations that best explains the analyzed dataset, we performed two approaches. These tools focus on the minimization of sources within the variation of the group. Therefore, we used Principal Component Analysis (PCA) and Discriminant Analysis of Principal Component (DAPC).

PCA is a statistical method used to simplify a multivariable dataset, with minimal loss of information. This technique uses Euclidean distance as a measure of dissimilarity and can be used to emphasize variation and bring out strong patterns in a dataset [33].

DAPC uses a k-means clustering algorithm and a Bayesian Inference Criterion (BIC) to determine the number of population groups (K), optimizing variation between groups and minimizing variation within groups [34].

Adegenet package in R software was used to perform PCA and DAPC.

## Results

Forty-five DNA samples extracted from sand flies specimens representing all Brazilian regions were PCR amplified for three gene fragments (period gene, IVS6 from cacophony gene and 12SrDNA). In total, 103 loci of nine populations were evaluated (BioProject ID: PRJNA555630). After processing the sequencing data, we observed that in 3 specimens (1 from São Borja, 1 from Fortaleza and 1 from Palmas) we obtained very few sequences for at least one of the amplified fragments (period gene, IVS6 from cacophony gene and 12SrDNA), for this reason we decided to exclude them for further analysis.

Estimates of FST pairwise were not significant. However, these values revealed that the mean of genetic differentiation among populations ranged from 0 (0.000) to 5% (0.0500) (Table 3). Although the values do not indicate significant genetic differentiation, populations of *Lu*. *longipalpis* from Belém (PA) and Jequié (BA) were genetically closer, whereas populations from São Borja (RS) and Fortaleza (CE) were most genetically distinct.

**Table 3:** Estimates of FST pairwise of *Lu*. *longipalpis* populations from nine municipalities of Brazil.

	**Campo** **Grande**	**Miranda**	**São** **Borja**	**Governador** **Valadares**	**Jequié**	**Palmas**	**Belém**	**Recife**	**Fortaleza**
**Campo** **Grande**	-	-	-	-	-	-	-	-	-
**Miranda**	0.0076	-	-	-		-	-	-	-
**São** **Borja**	0.0104	0.0095	-	-	-	-	-	-	-
**Governador** **Valadares**	0.0192	0.0303	0.0327	-	-	-	-	-	-
**Jequié**	0.0277	0.0330	0.0418	0.0101	-	-	-	-	-
**Palmas**	0.0139	0.0256	0.0283	0.0113	0.0095	-	-	-	-
**Belém**	0.0186	0.0238	0.0337	0.0103	-0.0000	0.0044	-	-	-
**Recife**	0.0205	0.0289	0.0364	0.0231	0.0225	0.0192	0.0194	-	-
**Fortaleza**	0.0336	0.0440	0.0500	0.0377	0.0327	0.0282	0.0297	0.0005	-

Statistically significant correlations were detected between genetic differentiation and geographic distances based on the Mantel test [r(AB) = 0.9381417; p < 0.00001 e alpha = 0.05]. The results suggested that geographic distance significantly contributes to the genetic differentiation observed in the *Lu*. *longipalpis* populations.

**Fig 2:**
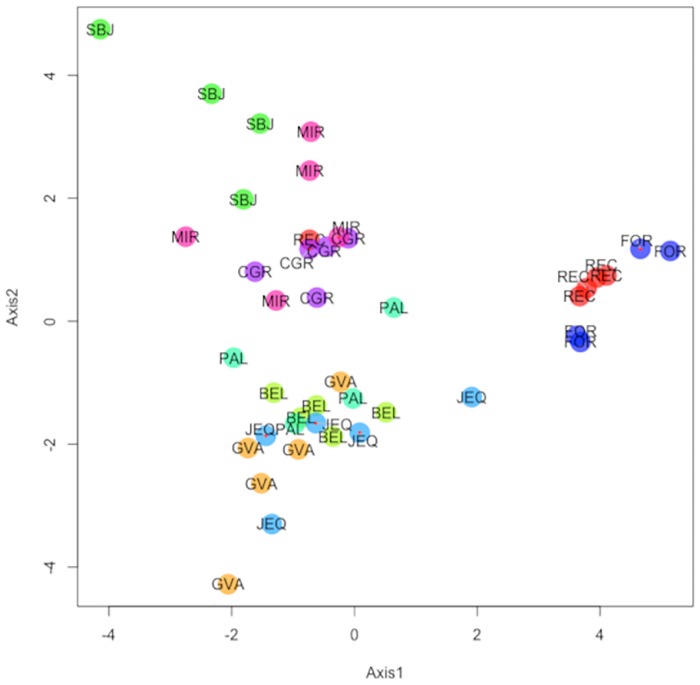
Principal Components Analysis (PCA) of *Lu*. *longipalpis* specimens from all regions of Brazil using data of 103 loci, obtained from three markers. REC: Recife; GVA: Governador Valadares; BEL: Belém; SOB: São Borja; PAL: Palmas; JEQ: Jequié; FOR: Fortaleza; CGR: Campo Grande; MIR: Miranda.

**Fig 3:**
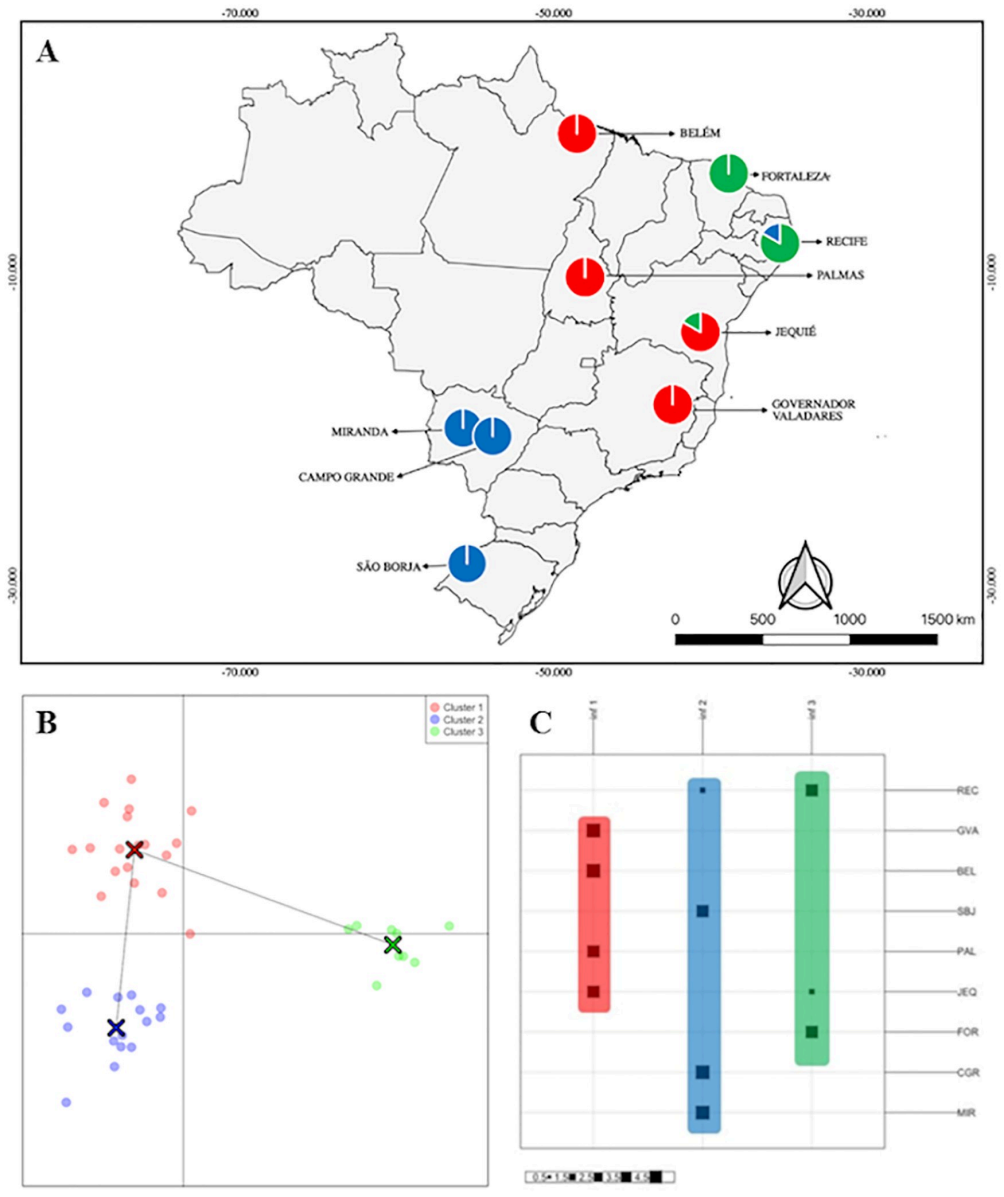
Principal Component Discriminant Analysis (DAPC) of *Lu*. *longipalpis* populations from all regions of Brazil using data of 103 loci, obtained from three markers. (A) Pie charts of the clusters assignment distribution in nine municipalities of Brazil plotted in a map. (B) Ordination of the clusters in two axes. Colors represent genetic clusters (blue, red, green). (C) Rows correspond to actual groups (n = 9), while columns correspond to inferred groups (n = 3). Square sizes represent numbers of individuals in each inferred cluster for the nine populations as depicted in the scale down below the figure.

We obtained similar results depending on the approaches used to estimate the clusters, considering that PCA and DAPC revealed three clusters (Figs 2 and 3).

## Discussion

Studies about the genetic structure of natural populations of vector insects have many applications in evolutionary biology and conservation, an essential aspect to understand the ecoepidemiology of the diseases [35]. Due to the importance of *Lu*. *longipalpis* in the epidemiological chain of visceral leishmaniasis, the knowledge about the taxonomic status of this species has increased considerably in recent years, producing several studies in Latin American countries [36].

Over the past 30 years, more than 100 articles have been published using molecular markers to relate or separate sand flies populations, species, groups or genera [36]. Different methodologies have been used, isolated or integrated, such as biochemical, morphological and molecular studies [37, 38, 39, 40]. Some molecular approaches have been used in order to investigate these intra and interspecific variations: Restriction Fragment Length Polymorphism (RFLP), DNA hybridization, Random Amplification of Polymorphic DNA (RAPD-PCR), Single Strand Conformation Polymorphism (SSCP), microsatellites, sequencing of the mitochondrial and nuclear genome [17, 36, 37, 41, 42, 43, 44, 45].

The increase of the molecular studies has encouraged the enhancement of DNA sequencing technologies. One of the most relevant approaches within ecological and population genetics was the development of *high-throughput* genotyping (Next Generation Sequencing—NGS). This technique allows the analysis of large-scale molecular markers, as well as improving inferences about the genetic variability of populations [46], kinship attributions [47] and understanding of historical demographic patterns and introgression events [48].

There is much interest in applying NGS for sequencing directed to specific genes and in large numbers of individuals [49]. Considering the decreasing sequencing costs and increased molecular markers studies, Golczer and Arrivillaga [36] suggested that researchers should use more than one molecular marker to understand genetic and evolutionary issues greater robustness of the result. Therefore, we investigated genetic diversity in nine *Lu*. *longipalpis* populations, using the sequencing with the Illumina MiSeq platform, in three markers, simultaneously.

It is noteworthy that our sequences generated by the Illumina MiSeq platform of each individual are the product of more than a thousand direct and reverse sequences per fragment. Thus, possible sequence interpretation errors could be detected and filtered, since thousands of sequences are considered. It was different if we used Sanger’s sequencing when only a single sequence is present. Shokralla et al. [50] compared the investment cost and total time spent between the Sanger and Illumina Miseq processes. They observed a 27% reduction in total time and 79% reduction in labor costs using NGS. This cost reduction enables the development of a larger number of projects with the same value in a single survey.

Estimating genetic differentiation among populations (FST) is fundamental in genetic studies to understand population demographic history [51]. Our results demonstrate low FST pairwise values (Table 3) indicating a low level of genetic differentiation among *Lu*. *longipalpis* subpopulations, according to the classification proposed by Wright [52], Hart and Clark [53].

Genetic differentiation results from several evolutionary processes, such as gene flow, natural selection, and isolation by geographic distance. The last one is a vicarious process capable of causing speciation in different populations of the same species. This event occurs because geographic space leads to environmental variation [54, 55, 56] and, with increasing distance, gene flow attenuates and tends to cease [57, 58].

We used the Mantel test in order to evaluate the hypothesis that the levels of population structure of *Lu*. *longipalpis* could be associated with distance isolation. This test showed a significant (p<0.00001) positive correlation (r(AB) = 0.9381417) between the genetic distances (FST) and geographic (Km). This result shows that, although there was little genetic differentiation between the individuals, there is a strong isolation by distance between the nine analyzed populations that covered a distance variation of up to 3,334 km.

Our results confirm a study of genotyping based on microsatellites, which showed Brazilian populations of *Lu*. *longipalpis* genetically divergent, consistent with geographic distance [37]. Based on the mitochondrial gene ND4, Soto et al. [31] also observed a strong correlation between the geographic distances in the genetic differentiation among the Honduran populations of *Lu*. *longipalpis*, suggesting that this fact is due to the limited capacity of locomotion of the sandflies and caused by the innumerable geographic and climatic barriers that could limit or even prevent gene flow among populations. This hypothesis may explain the distance isolation found in our results, considering that the specimens were collected at geographic distances higher than the study by Soto et al. [31]. Furthermore, Brazil is a country with continental dimensions and the specimens were collected in different biomes and, consequently, in different ecological conditions.

Evaluating the isolation by geographic distance is fundamental. This phenomenon causes, through the time, a new characteristic in one of the populations like, for example, a new sound of courtship, causing the break of the gene flow [59]. This event can lead to important epidemiological and ecological consequences, such as changes in vector capacity and competence, resistance to insecticides and limit/prevent/retard the speciation process [60].

*Lutzomyia longipalpis* populations analyzed in this study were submitted to PCA to verify possible clusters by similarity of frequencies of the examined alleles. Jombart, Devillard and Balloux [61] argue that this method does not have some essential characteristics to investigate the genetic structure of biological populations because it does not provide a group assessment and does not require an *a priori* definition of clusters to study population structures. However, PCA has been widely used for genetic analysis as an alternative to Bayesian clustering algorithms. In addition, PCA (Fig 2) provided a clear separation between the different *Lu*. *longipalpis* populations.

Due to the possibility of bias introduced by the absence of *a priori* cluster determination using PCA, we still use another approach to confirm possible clustering. Observing DAPC (Fig 3A, 3B and 3C), *Lu*. *longipalpis* populations were also grouped into three distinct clusters (k = 3). This analysis confirmed the clusters demonstrated by the PCA, validating its results. The use of different approaches to analyzing clusters in population genetics is extremely important, providing less biased data evaluation. An advantage of DAPC in relation to other clustering approaches is the possibility of generating a graphical representation of the relationship between inferred clusters [61], as observed in Fig 3C.

Brazilian populations of *Lu*. *longipalpis* present morphological variation and differ in the number of spots present in the abdominal tergites, a characteristic observed by Mangabeira [11], when he was studying specimens collected in the States of Ceará and Pará. The specimens from the state of Pará (PA) had a pair of pale spots on the IV abdominal tergite (phenotype called one spot—'1S'), while those from the state of Ceará (CE) had two pairs of spots (the phenotype two spot—'2S'), one in the IV tergite and one in the tergite III. Mangabeira also mentioned that the two forms were found in different ecological conditions, speculating that they could represent different species or a variety of them. Subsequently, intermediate phenotypes (a pair of pale spots with a minor point on tergite III) were observed, indicating an intraspecific polymorphism [38, 39].

In the DAPC (Fig 3), the populations analyzed in this study formed clusters similar to the phenotypes (morphotypes 1S and 2S). In the red cluster, *Lu*. *longipalpis* populations were grouped morphotype 1S of the North and Northeast of the country: Palmas (TO), Belém (PA), Jequié (BA) and Governador Valadares (MG). In the green cluster, *Lu*. *longipalpis* populations were grouped of the Northeast morphotype 2S of Recife and Fortaleza. Finally, in the blue cluster, *Lu*. *longipalpis* specimens were grouped of the Midwest and South of the country—Campo Grande (MS), Miranda (MS) and São Borja (RS). *Lutzomyia longipalpis* populations of Miranda (MS) and São Borja (RS) present morphotype 1S, while the population of Campo Grande was composed by specimens morphotypes 1S and 2S.

Molecular studies presuppose that *Lu*. *longipalpis* populations 1S (one spot) and 2S (two spot) are in recent process of speciation (0.22 to 1.02 million years ago) [62, 63]. This period is believed that was sufficient to generate morphological diversity and to create the new species, *Lu*. *pseudolongipalpis* [64], which is possibly occurring among the different *Lu*. *longipalpis* populations.

In addition to phenotypic differences, a behavioral aspect studied is the production of “*lovesongs*” by *Lu*. *longipalpis* males during mating [65, 66, 67]. The reproductive isolation observed among Brazilian *Lu*. *longipalpis* populations are caused by failures during intercourse between specimens that produce different types of copulatory *lovesongs*. This acoustic signal may play an important role in species recognition, acting as a reproductive barrier and reducing gene flow [40, 65, 66]. These *lovesongs* are controlled by genes such as *period* (*per*) and *cacophony* (*cac*). These genic regions are analyzed in the present study, since they play an important role in species recognition.

Beyond the phenotypic and behavioral differences found in the Brazilian populations of *Lu*. *longipalpis*, there are also differences considering the main component of sex pheromones. In Brazil, the populations of *Lu*. *longipalpis* produce four different chemotypes (9-methylgermacrene-B, 3-methyl-α-himachalene, cembrene-1 and cembrene-2)[39, 68]. This fact is relevant since there is evidence that members of the same species that produce different sex pheromones are reproductively isolated [68]. In Fig 3, we can observe that the distribution of the clusters in our results coincides with the location of the different sex pheromone chemotypes of *Lutzomyia longipalpis* in Brazil presented in the Spiegel et al. [39] research. *Lu*. *longipalpis* populations of Recife (PE) and Fortaleza (CE) produce cembrene-1, whereas the populations of São Borja (RS), Campo Grande (MS) and Miranda (MS) produce 9-methylgermacrene-B. This fact could justify these clusters found in our results and be the factor responsible for the reproductive isolation of the species.

Studying complex of species, such as *Lu*. *longipalpis*, we used genetic markers directly involved in the speciation process. Therefore, we chose to amplify and analyze two nuclear regions: the IVS6 region of the *cacophony* gene and the *period* gene. The IVS6 region of the *cacophony* gene encodes the α-1 subunit of a voltage-dependent calcium channel. While the gene *period* is involved in the circadian rhythm and controls the species-specific differences in locomotor and mating activities [18, 19, 21].

The results obtained in studies of *Lu*. *longipalpis* complex using the *per* gene are consistent with those obtained in studies of pheromones and copulatory *lovesongs* [12]. Bauzer et al. [18] identified results that pointed to the existence of a species complex, since polymorphisms of the *per* gene may result in reproductive isolation.

Lins et al. [25] demonstrated that the IVS6 region of the *cac* gene can be used as an excellent molecular marker in population genetics studies, because this genomic region presents an intron with high variability and divergence between species. Bottechia et al. [19] observed that this region shows greater variability of gene flow than the *per* gene, suggesting that the IVS6 region may be more affected by introgression.

Another target of studies has been the mtDNa, as the 12S region, to evaluate the differentiation of populations of the *Lu*. *longipalpis* complex. The mtDNA presents maternal origin, evolves rapidly and does not recombine, being an adequate target to trace genealogy and evolutionary history. The 12S region of mtDNA was used by Beati et al. [24] when analyzing genetically different species of the genus *Lutzomyia* of Colombia and Peru, and identified different species of the genus in those countries.

In our conception and according to our results, the claim of Souza, Brazil and Araki [38] is correct when they affirm that there is no more doubt that the different populations of *Lu*. *longipalpis* belong to a complex of species. This premise can be carry out after analyzing morphological and sexual pheromone studies [15, 16, 39, 68, 69], copulatory *lovesongs* [12, 40, 44, 65, 66, 70] and molecular analyzes [36, 37, 41, 42, 44, 45, 71].

## Conclusions

*Lutzomyia longipalpis* genetic structure showed similar patterns according to the approach used, since both PCA and DAPC identified three populations. Thus, the use of different approaches to analyze clusters in population genetics was useful to provide a less biased data evaluation.

Studies about the *Lu*. *longipalpis* complex genetic structures can provide details on population differentiation and contribute to understand the processes of divergence and speciation, mechanisms responsible for the heterogeneity of vector capacity and competence, as well as vector susceptibility to infectious agents or insecticides. Thus, the evaluation of the population genetics of this vector can help to plan control measures appropriate to the real conditions of each transmission area of ​​this important endemic in public health.

## References

[1] Lutz A, Neiva A. Contribuição para o conhecimento das espécies do gênero *Phlebotomus* existente no Brasil. Memórias do Instituto Oswaldo Cruz. 1912; 4(1): 84–95. 10.1590/S0074-02761912000100006

[2] Deane LM, Deane MP. Encontro de leishmânias nas vísceras e na pele de uma raposa em zona endêmica de calazar, nos arredores de Sobral. Hospital. 1954a; 45: 419–421. 13183549

[3] Deane MP, Deane LM. Infecção natural do *Phlebotomus longipalpis* por leptomonas, provavelmente de *Leishmania donovani*, em foco de calazar, no Ceará. Hospital. 1954b; 45: 697–702. 13191739

[4] Deane LM. Leishmaniose Visceral no Brasil. Estudos sobre reservatórios e Transmissores realizados no estado do Ceará. Serviço Nacional de Educação Sanitária, Brasil.1956; viii: 82–84.

[5] Lainson R, Ward RD, Shaw JJ. Experimental transmission of *Leishmania chagasi* causative agent of neotropical visceral leishmaniasis, by the sandfly *Lutzomyia longipalpis*. Nature. 1977; 266: 628–630. 10.1038/266628a0 859627

[6] Lainson R, Rangel EF. *Lutzomyia longipalpis* and the eco-epidemiology of American visceral leishmaniasis, with particular reference to Brazil–a review. Memórias do Instituto Oswaldo Cruz. 2005; 100: 811–827. 10.1590/s0074-02762005000800001 16444411

[7] WHO. World Health Organization. Leishmaniasis. Fact sheet. 2017. http://www.who.int/mediacentre/factsheets/fs375/en/

[8] Aguiar GM, Medeiros WM. Distribuição regional e habitats das espécies de flebotomíneos do Brasil In: RANGEL E.; LAINSON R. Flebotomíneos do Brasil. Rio de Janeiro: Fiocruz, cap.3, p.207–255, 2003.

[9] Salomón OD, Basmajdian Y, Fernández MS, Santini MS. *Lutzomyia longipalpis* in Uruguay: the first report and the potential of visceral leishmaniasis transmission. Memórias do Instituto Oswaldo Cruz. 2011; 106: 381–382. 10.1590/s0074-02762011000300023 21655832

[10] Souza GD, Santos E, Andrade-Filho JD. The first report of the main vector of visceral leishmaniasis in America, *Lutzomyia longipalpis* (Lutz & Neiva) (Diptera: Psychodidae: Phlebotominae), in the state of Rio Grande do Sul, Brazil. Memórias do Instituto Oswaldo Cruz. 2009; 104: 1181–1182. 10.1590/s0074-02762009000800017 20140381

[11] Mangabeira O. Sobre a sistemática e Biologia dos Phlebotomus do Ceará. Revista Brasileira de Malariologia e Doenças Tropicais. 1969; 21: 3–25. 5385118

[12] Araki AS, Vigoder FM, Bauzer LGSR, Ferreira GEM, Souza NA, Araujo IB, et al Molecular and Behavioral Differentiation among Brazilian Populations of *Lutzomyia longipalpis* (Diptera:Psychodidae:Phlebotominae). Plos Neglected Tropical Diseases. 2009; 3: (e365): 1–12. 10.1371/journal.pntd.0000365.g001 PMC2628317 19172187

[13] Bauzer LGSR, Souza NA, Maingon RDC, Peixoto A. *Lutzomyia longipalpis* in Brazil: a complex or a single species? A mini-review. Memórias do Instituto Oswaldo Cruz. 2007; 102(1): 1–12. 10.1590/s0074-02762007000100001 17293992

[14] Gouveia C, Asensi MD, Zahner V, Rangel EF, Oliveira SMP. Study on the Bacterial Midgut Microbiota Associated to Different Brazilian Populations of *Lutzomyia longipalpis* (Lutz & Neiva) (Diptera: Psychodidae). Neotropical Entomology. 2008; 37: 597–601. 10.1590/S1519-566X2008000500016 19061048

[15] Hamilton JGC, Dougherty MJ, Ward RD. Sex pheromone activity in a single component of tergal gland extract of *Lutzomyia Longipalpis* (Diptera: Psychodidae) from Jacobina, Northeastern Brazil. Journal of Chemical Ecology. 1994; 2: 141–151. 10.1007/BF02065997 24241705

[16] Lane R, Phillips A, Procter G, Ward RD. Chemical analysis of the abdominal glands of two forms of *Lutzomyia longipalpis*: site of a possible sex pheromone. Annals of Tropical Medicine Parasitology. 1985; 79(2): 225–229. 10.1080/00034983.1985.11811912 4096569

[17] Aransay A, Scoulica E, Tselentis Y, Ready P. Phylogenetic relationships of phlebotomine sandflies inferred from small subunit nuclear ribosomal DNA. Insect Molecular Biology. 2000; 9: 157–168. 10.1046/j.1365-2583.2000.00168.x 10762423

[18] Bauzer L, Gesto J, Souza N, Ward R, Hamilton J, Kyriacou C, et al Molecular divergence in the period gene between two putative sympatric species of the *Lutzomyia longipalpis* Complex. Molecular Biology and Evolution. 2002; 19: 1624–1627. 10.1093/oxfordjournals.molbev.a004224 12200489

[19] Bottechia M, Oliveira SG, Bauzer LGSR, Souza NA, Ward RD, Garner KJ, et al Genetic divergence in the cacophony IVS6 intron among five Brazilian populations of *Lutzomyia longipalpis*. Journal of Molecular Evolution. 2004; 58: 754–761. 10.1007/s00239-004-2586-y 15461432

[20] Depaquit J, Perrotey S, Lecointre G, Tillier A, Tillier S, Ferté H, et al Systématique moléculaire des phlebotominae: Étude pilote. Paraphylie du genre *Phlebotomus*. Comptes Rendus de l’Académie des Sciences. 1998; 321: 849–855. 10.1016/s0764-4469(99)80025-0 9835021

[21] Lins RM, Souza NA, Peixoto AA. Genetic divergence between two sympatric species of the *Lutzomyia longipalpis* complex in the paralytic gene, a locus associated with insecticide resistance and lovesong production. Memórias do Instituto Oswaldo Cruz. 2008; 103: 736–740. 10.1590/s0074-02762008000700019 19057828

[22] Ribolla PEM, Gushi LT, Cruz MSP, Costa CHN, Costa DL, Lima-Jr MSC, et al *Leishmania infantum* Genetic Diversity and Lutzomyia longipalpis Mitochondrial Haplotypes in Brazil. BioMed Research International. 2016; Article ID 9249217: 1–11. 10.1155/2016/9249217 27119085 PMC4828539

[23] Galati EAB. Classification Morphology and Terminology of Adults and Identification of American Taxa In: Rangel EF, Lainson R, editors. Brazilian Sand Flies: Biology, Taxonomy, Medical Importance and Control. Springer; 2018 pp. 9–212.

[24] Beati L, Cáceres A, Lee J, Munstermann L. Systematic relationships among *Lutzomyia* sand flies (Diptera: Psychodidae) of Peru and Colombia based on the analysis of 12S and 28S ribosomal DNA sequences. International Journal for Parasitology. 2004; 34: 225–234. 10.1016/j.ijpara.2003.10.012 15037108

[25] Lins RMMA, Oliveira SG, Souza NA, Queiroz RG, Justiniano SCB, Ward RD, et al Molecular evolution of the cacophony IVS6 region in sandflies. Insect Molecular Biology. 2002; 11: 117–122. 10.1046/j.1365-2583.2002.00315.x 11966876

[26] Langmead B, Trapnell C, Pop M, Salzberg SL. Ultrafast and memory-efficient alignment of short DNA sequences to the human genome. Genome Biology. 2009; 10: R25.1–R25. 10.1186/gb-2009-10-3-r25 19261174 PMC2690996

[27] Weir BS, Cockerham CC. Estimating F-statistics for the analysis of population structure. Evolution. 1984; 38:1358–1370. 10.1111/j.1558-5646.1984.tb05657.x 28563791

[28] Coutinho-Abreu IV, Sonoda IV, Fonseca JA, Melo MA, Balbino VQ, Ramalho-Ortigão M. *Lutzomyia longipalpis* s.l. in Brazil and the impact of the Sao Francisco River in the speciation of this sand fly vector. Parasites & Vectors. 2008; 1: 1–11. 10.1186/1756-3305-1-16 18549496 PMC2474595

[29] Ebrahimi S, Bordbar A, Parvizi P. Genetic dynamics in the sand fly (Diptera: Psychodidae) nuclear and mitochondrial genotypes: evidence for vector adaptation at the border of Iran with Iraq. Parasites & Vectors. 2016; 9: 1–13. 10.1186/s13071-016-1603-5 27260204 PMC4893242

[30] Mantel N. The detection of disease clustering and a generalized regression approach. Cancer Research. 1967; 27: 209–220. 6018555

[31] Soto SIU, Lehmann T, Rowton ED, Vélez ID, Porter CH. Speciation and Population Structure in the Morphospecies *Lutzomyia longipalpis* (Lutz & Neiva) as Derived from the Mitochondrial ND4 Gene. Molecular Phylogenetics and Evolution. 2001; 18: 84–93. 10.1006/mpev.2000.0863 11161745

[32] Zhang L, Ma Y, Xu J. Genetic differentiation between sandfly populations of *Phlebotomus chinensis* and *Phlebotomus sichuanensis* (Diptera: Psychodidae) in China inferred by microsatellites. Parasites & Vectors. 2013; 6: 1–10. 10.1186/1756-3305-6-115 23607337 PMC3649936

[33] Hongyu K, Sandanielo VLM, Oliveira GJ Jr. Análise de Componentes Principais: resumo teórico, aplicação e interpretação.—Engineering and Science. 2015; 5: 83–90.

[34] Ogawa LM, Vallender EJ. Genetic substructure in cynomolgus macaques (*Macaca fascicularis*) on the island of Mauritius. BMC Genomics. 2014; 15:1–14. 25174998 10.1186/1471-2164-15-748 PMC4167525

[35] Meirmans PG, Hedrick PW. Assessing population structure: FST and related measures. Molecular Ecology Resources. 2011; 11: 5–18. 10.1111/j.1755-0998.2010.02927.x 21429096

[36] Golczer G, Arrivillaga J. Use and trends of molecular markers in sandflies (Diptera: Psychodidae). Boletin de Malariologia y Salud Ambiental. 2015; 55:19–40.

[37] Santos MFC, Ribolla PEM, Alonso DP, Andrade-Filho JD, Casaril AE, Ferreira AMT, et al Genetic structure of *Lutzomyia longipalpis* populations in Mato Grosso do Sul, Brazil, based on microsatellite markers. Plos One. 2013; 8:1–7. 10.1371/journal.pone.0074268 24066129 PMC3774819

[38] Souza NA, Brazil RP, Araki AS. The current status of the *Lutzomyia longipalpis* (Diptera: Psychodidae: Phlebotominae) species complex. Memórias do Instituto Oswaldo Cruz. 2017; 112: 161–174. 10.1590/0074-02760160463 28225906 PMC5319373

[39] Spiegel CN, Dias DBS, Araki AS, Hamilton JGC, Brazil RP. Jones TM. The *Lutzomyia longipalpis* complex: a brief natural history of aggregation-sex pheromone communication. Parasites & Vectors. 2016; 9 10.1186/s13071-016-1866-x 27842601 PMC5109651

[40] Vigoder FM, Souza NA, Brazil RP, Bruno RV, Costa PL, Ritchie MG, et al Phenotypic differentiation in love song traits among sibling species of the *Lutzomyia longipalpis* complex in Brazil. Parasites & Vectors. 2015; 8 10.1186/s13071-015-0900-8 26017472 PMC4456791

[41] Adamson RE, Ward RD, Feliciangeli MD. Maingon R. The application of random amplified polymorphic DNA for sandfly species identification. Medical and Veterinary Entomology. 1993; 7: 203–207. 10.1111/j.1365-2915.1993.tb00677.x 8369553

[42] Arrivillaga J, Mutebi J, Piñango H, Norris D, Alexander B, Feliciangeli M, et al The taxonomic status of genetically divergent populations of *Lutzomyia longipalpis* (diptera: Psychodidae) based on the distribution of mitochondrial and isozyme variation. Journal of Medical Entomology. 2003; 40: 615–627. 10.1603/0022-2585-40.5.615 14596274

[43] Maingon R, Feliciangeli MD, Ward R, Chance M, Adamson R, Rodriguez N, et al Molecular approaches applied to the epidemiology of leishmaniasis in Venezuela. Archives De l’Institut Pasteur De Tunis. 1993; 70: 309–324. 7802485

[44] Maingon R, Ward R, Hamilton J, Noyes H, Souza N, Kemp S, et al Genetic identification of two sibling species of *Lutzomyia longipalpis* (Diptera: Psychodidae) that produce distinct male sex pheromones in Sobral, Ceará state, Brazil. Molecular Ecology. 2003; 12: 1879–1894. 10.1046/j.1365-294x.2003.01871.x 12803639

[45] Vivero R, Contreras-Gutierrez M, Bejarano E. Análisis de la estrutura primaria y secundaria del ARN de transferência mitocondrial para serina em siete especies de *Lutzomyia*. Biomédica. 2007; 27: 429–438. 18320108

[46] Lozier JD. Revisiting comparisons of genetic diversity in stable and declining species: assessing genome-wide polymorphism in North American bumble bees using RAD sequencing. Molecular Ecology. 2014; 23: 788–801. 10.1111/mec.12636 24351120

[47] Novembre J, Stephens M. Interpreting principal component analyses of spatial population genetic variation. Nature Genetics. 2008; 40: 646–649. 10.1038/ng.139 18425127 PMC3989108

[48] Ekblom R, Galindo J. Applications of next generation sequencing in molecular ecology of non-model organisms. Heredity. 2011; 107:1–15. 10.1038/hdy.2010.152 21139633 PMC3186121

[49] Harismendy O, Ng PC, Strausberg RL, Wang X, Stockwell TB, Beeson KY, et al Evaluation of next generation sequencing platforms for population targeted sequencing studies. Genome biology.2009; 10: R32.1–32.13. 10.1186/gb-2009-10-3-r32 19327155 PMC2691003

[50] Shokralla S, Porter TM, Gibson JF, Dobosz R, Janzen DH, Hallwachs W, et al Massively parallel multiplex DNA sequencing for specimen identification using an Illumina MiSeq platform. Scientific Reports. 2015; 5: 1–7. 10.1038/srep09687 25884109 PMC4401116

[51] Willing EM, Dreyer C, Oosterhout C. Estimates of Genetic Differentiation Measured by FST Do Not Necessarily Require Large Sample Sizes When Using Many SNP Markers. Plos One. 2012; 7: e42649–e42649. 10.1371/journal.pone.0042649 22905157 PMC3419229

[52] Wright S. Evolution and Genetics of Populations: Variability within and among Natural Populations. University of Chicago Press; 1978.

[53] Hart DL, Clark AG. Princípios de Genética de Populações. 4st ed Editora Artmed; 2010.

[54] Darwin C. On the origin of species. The Eletronic Classic Series Hazleton; 1859.

[55] Polato NR, Gray MM, Gill BA, Becker CG, Casner KL, Flecker AS, et al Genetic diversity and gene flow decline with elevation in montane mayflies. Heredity. 2017; 119: 107–116. 10.1038/hdy.2017.23 28489073 PMC5520546

[56] Star B, Spencer HG. Effects of Genetic Drift and Gene Flow on the Selective Maintenance of Genetic Variation. Genetics. 2013; 194: 235–244. 10.1534/genetics.113.149781 23457235 PMC3632471

[57] Mayr E. Systematics and the Origin of Species, from the Viewpoint of a Zoologist. Columbia University Press; 1942.

[58] Martins AB. Especiação por distância e a evolução de espécies em anel. PhD Thesis—Instituto de Biociências da Universidade de São Paulo. Departamento de Ecologia, 2014. http://www.teses.usp.br/teses/disponiveis/41/41134/tde-09122014-152748/pt-br.php.

[59] Ridley M. Evolução. 3 st ed. Editora Artmed; 2006.

[60] Slatkin M. Gene Flow and the Geographic Structure of Natural Populations. Science. 1987; 236: 787–792. 10.1126/science.3576198 3576198

[61] Jombart T, Devillard S, Balloux F. Discriminant analysis of principal components: a new method for the analysis of genetically structured populations. BMC Genetics. 2010; 11: 1–15. 20950446 10.1186/1471-2156-11-94 PMC2973851

[62] Andrade-Filho JD, Brazil RP. Relationships of New World Phlebotomine Sand Flies (Diptera: Psychodidae) Based on Fossil Evidence. Memórias do Instituto Oswaldo Cruz. 2003; 98: 145–149. 10.1590/S0074-02762003000900022 12687775

[63] Watts PC, Hamilton J G, Ward RD, Noyes HA, Souza NA, Kemp SJ, et al Male sex pheromones and the phylogeographic structure of the *Lutzomyia longipalpis* species complex (Diptera: Psychodidae) from Brazil and Venezuela. The American Journal of Tropical Medicine and Hygiene. 2005; 73: 734–743. 16222018

[64] Arrivillaga JC, Feliciangeli MD. *Lutzomyia pseudolongipalpis*: The First New Species Within the longipalpis (Diptera: Psychodidae: Phlebotominae) Complex from La Rinconada, Curarigua, Lara State, Venezuela. Entomological Society of America. 2001; 38: 783–790. 10.1603/0022-2585-38.6.783 11761375

[65] Souza NA, Ward RD, Hamilton JGC, Kyriacou CP, Peixoto AA. Copulation songs in three siblings of *Lutzomyia longipalpis* (Diptera: Psychodidae). Transactions of the Royal Society of Tropical Medicine and Hygiene. 2002; 96: 102–103. 10.1016/s0035-9203(02)90258-0 11925981

[66] Souza NA, Vigoder FM, Araki AS, Ward RD, Kyriacou CP, Peixoto AA. Analysis of the copulatory courtship songs of *Lutzomyia longipalpis* in six populations from Brazil. Journal of Medical Entomology. 2004; 41: 906–913. 10.1603/0022-2585-41.5.906 15535620

[67] Ward R, Phillips A, Burnet B, Marcondes C. The *Lutzomyia longipalpis* complex: reproduction and distribution In Biosystematics of Haematophagous Insects. Oxford: Oxford University Press 1988; 258–69.

[68] Hamilton JGC, Maingon RDC, Alexander B, Ward RD, Brazil RP. Analysis of the sex pheromone extract of individual male *Lutzomyia longipalpis* sandflies from six regions In Brazil. Medical and Veterinary Entomology. 2005; 19: 480–488. 10.1111/j.1365-2915.2005.00594.x 16336313

[69] Hamilton JGC, Dawson GW, Pickett JA. 9-Methylgermacrene-B; proposed structure for the novel homosesquiterpene sex pheromone of *Lutzomyia longipalpis* (Diptera: Psychodidae) from Lapinha, Brazil. Journal of Chemical Ecology. 1996; 22: 1477–1492. 10.1007/BF02027726 24226250

[70] Vigoder FM, Araki AS, Bauzer LGSR, Souza NA, Brazil RP, Peixoto AA. Lovesongs and period gene polymorphisms indicate *Lutzomyia cruzi* (Mangabeira, 1938) as a sibling species of the *Lutzomyia longipalpis* (Lutz and Neiva, 1912) complex. Infection, Genetics and Evolution. 2010; 10: 734–739. 10.1016/j.meegid.2010.05.004 20478408

[71] Aransay A, Scoulica E, Chaniotis B, Tselentis Y. Typing of sandflies from Greece and Cyprus by DNA polymorphism of 18S rRNA gene. Insect Molecular Biology. 1999; 8: 179–184. 10.1046/j.1365-2583.1999.820179.x 10380101

